# Natural history of isolated abdominal aortic dissection: A prospective cohort study

**DOI:** 10.3389/fcvm.2023.1002832

**Published:** 2023-02-23

**Authors:** Jinlin Wu, Yanfen Wu, Fei Li, Donglin Zhuang, Yunqing Cheng, Zerui Chen, Jue Yang, Jie Liu, Xin Li, Ruixin Fan, Tucheng Sun

**Affiliations:** ^1^Department of Cardiac Surgery, Guangdong Cardiovascular Institute, Guangdong Provincial People’s Hospital, Guangdong Academy of Medical Sciences, Guangzhou, China; ^2^Department of Cardiac Surgery, Beijing Anzhen Hospital, Capital Medical University, Beijing, China

**Keywords:** isolated abdominal aortic dissection, natural history, survival, treatment, aortic

## Abstract

**Objectives:**

Isolated abdominal aortic dissection (IAAD) is extremely rare, with its optimal treatment and intervention timing remaining poorly understood. We aimed to study the natural history of IAAD and facilitate better clinical decision.

**Methods:**

Consecutive patients admitted to our institution from January 2016 to April 2021 were enrolled and followed up prospectively. All-cause death was taken as the primary endpoint.

**Results:**

A total of 68 patients with IAAD were included. The mean age at presentation was 61.2 ± 14.8 (Range: 26.0, 93.0) years and 55 (80.9%) were male. A total of 38 (55.9%) patients were treated conservatively, 27 (39.7%) received endovascular aneurysm repair (EVAR), and 3 (4.4%) underwent open surgery. After a mean follow-up of 2.4 years (Range: 0.1, 5.5), 9 (13.2%) patients died, 8 of whom (21.0%) were treated conservatively and 1 EVAR (3.7%). Compared with EVAR/open surgery, patient treated conservatively had a much worse survival (*p* = 0.043). There was no significant difference between different IAAD aortic sizes regarding mortality (*p* = 0.220). Patients with completely thrombosed false lumen fared improved survival rate, followed by partial thrombosis and patency, respectively, although not significantly (*p* = 0.190). No significant difference was observed between male and female concerning survival rate (*p* = 0.970). Patients without symptoms had a significantly improved survival (*p* = 0.048).

**Conclusion:**

On the basis of patients’ preference and surgeons’ experience, a more aggressive treatment regimen for IAAD should be considered, with EVAR being the first choice, especially for those with persistent symptoms and patent false lumen, regardless of sex, age, or aortic size.

## Introduction

**Figure 1:**
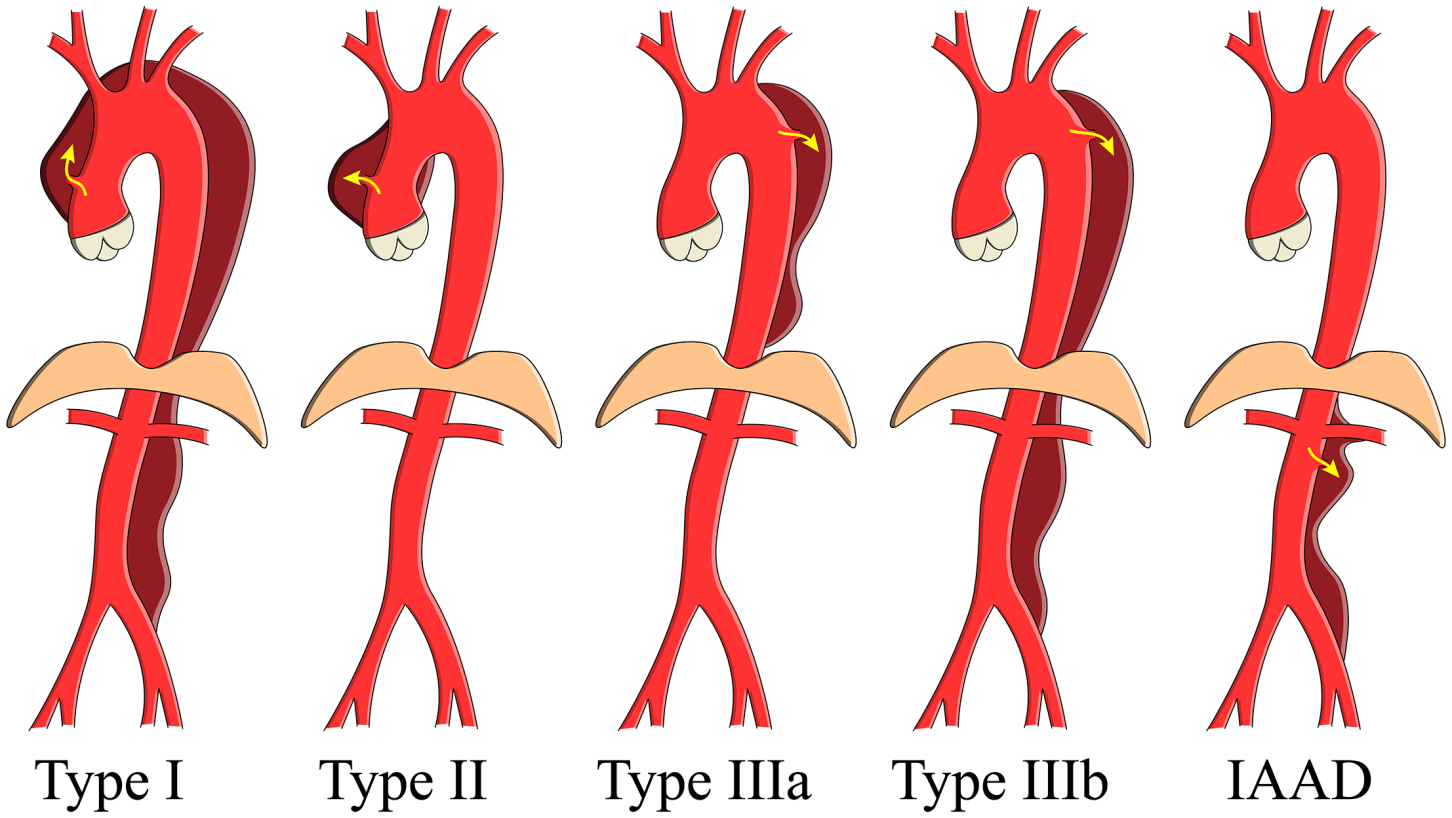
Schema showing the aortic dissection classification. IAAD, isolated abdominal aortic dissection.

Aortic dissection (AD) is a notorious killer and is often known as Stanford type A or B (1). Isolated abdominal aortic dissection (IAAD), referring to AD limited to the abdominal aorta, is believed to be a unique entity (Figure 1). We have performed a meta-analysis enrolled only 491 IAAD patients worldwide previously, and the incidence is estimated to be about 5.1/1,000,000 per year, which is extremely rare (2). Currently, the best treatment modality for IAAD, i.e., conservative or interventional [open surgery (OS) or endovascular aneurysm repair (EVAR)], remains controversial. For the timing of surgery, a natural history study is essential to select patients with a reasonable benefit to risk ratio for surgery, which is the key for both the doctor and patients to make a decision.

Unfortunately, due to the rarity of the disorder, there is a paucity of solid data on the natural history of IAAD. Importantly, Sen et al. (3) recently conducted a preliminary study of the natural history of IAAD. They found that the overall mortality of IAAD is similar to population controls. Unfortunately, as they pointed out that clinical recommendations or conclusions were hard to make with only 14 patients enrolled. In the present study, we aimed to study the natural history of IAAD with a relatively larger cohort, facilitating better clinical decision and providing more insights into this intriguing disorder.

## Methods

The prospective cohort study was reported in line with Strengthening the Reporting of Observational Studies in Epidemiology (STROBE) (4). The study was approved by the Institutional Review Board (KY-Q-2021-073-01), with informed consent not required due to its observational nature.

All consecutive patients admitted to the Guangdong Provincial People’s Hospital (Guangdong, China) from January 2016 to April 2021 were enrolled and followed up prospectively. Anthropometric, radiologic, laboratory, and operative data were manually accrued from individual electronic medical records and hospital charts. If there were missing values, we would check with the patient or relatives by phone. Computed Tomography Angiography (CTA) was used to confirm IAAD, demonstrating dissected intimal flap and double-lumen aorta below the diaphragm, with or without visible entry tear. Hypertension was diagnosed according to medical history as blood pressure measured at 140/90 mmHg or higher. Diameter was measured perpendicular to the centerline at the different levels in an outer-to-outer manner, and the maximum was noted. The thrombosis status of false lumen was classified as complete thrombosis (CT), partial thrombosis (PT, concurrent presence of both flow and thrombus), and patency (P) proposed by Tsai et al. (5). Accidental identification of IAAD indicated that the disease was diagnosed by chance such as routinely physical examination or undergoing imaging not specifically for aortic disease. Those patients usually had no symptoms and the aortic dissection was in chronic phase (6).

There was a lack of recognized protocol for the optimal management of IAAD. Patients were treated either conservatively with best medical therapy (BMT), or aggressively with OS or EVAR, based on attending surgeon’s judgment and patients’ preference. All-cause death was taken as the primary endpoint and surgical intervention for BMT cohort as the secondary endpoint. Patients were followed up either with clinical visits or phone calls.

### Statistical analysis

And this study could be the largest prospective cohort investigating the natural history of IAAD based on our literature review. The potential variables were chosen based on expert opinion, clinical reasoning, availability, literature and plausibility without statistical pre-selection. Continuous variables were tested for normality distribution with the Kolmogorov–Smirnov test and were expressed as a mean with standard deviation (SD) and range (minimum, maximum). Categorical variables were presented as frequencies with percentages. We calculated the survival rate and freedom from death and intervention using the Kaplan–Meier (K-M) analytical method (“survival,” “survivalAnalysis,” and “survminer” packages in R) combined with the log-rank test. Univariable Cox proportional hazards regression was used to estimate the hazard ratios (HRs) with 95% confidence interval (CI). Loss of follow-up or end of the study period were treated as censors during the time-to-event analysis. R software (version 3.5.1) was used for data analysis. A two-tailed *p* < 0.05 indicated statistical significance.

## Results

A total of 68 patients with IAAD were included in this study. Baseline information of these patients was shown in Table 1. The mean age at presentation was 61.2 ± 14.8 (Range: 26.0, 93.0) years and 55 (80.9%) were male. 38 (74.5%) had hypertension and 4 (7.8%) was complicated with diabetes mellitus. Thirteen (25.5%) suffered from hyperlipidemia and 10 (19.6%) developed chronic obstructive pulmonary disease. The incidence of coronary artery disease was 7 (13.7%), which was the same to the incidence of renal insufficiency. Thirty (58.8%) had a history of smoking and 5 (9.8%) had gout. Three (5.9%) had a history of cardiovascular surgery, and 1 (2.0%) reported a family history of aortic disease. Interestingly, up to 22 (32.8%) IAAD were identified accidentally without any perceivable symptoms. Further, the baseline characteristics of the conservatively treated cohort was shown in Table 2. A total of 38 (55.9%) patients received BMT with surveillance, 27 (39.7%) received EVAR, and 3 (4.4%) received OS. After a mean follow-up of 2.4 years (Range: 0.1, 5.5), a total of 9 (13.2%) patients died, 8 of whom received BMT (21.0%) and 1 of whom received EVAR (3.7%). Three patients in the BMT cohort underwent EVAR at 1.3, 1.7, and 1.9 years of follow-up, respectively, and all survived.

**Table 1:** Baseline characteristics of the overall cohort.

Variables	Mean ± SD (Range)/Count (Percentage)
*n*	68
Age (year, mean ± SD)	61.2 ± 14.8 (Range: 26.0, 93.0)
Male (%)	55 (80.9)
Hypertension (%)	38 (74.5)
Diabetes (%)	4 (7.8)
Hyperlipidimia (%)	13 (25.5)
Chronic obstructive pulmonary disease (%)	10 (19.6)
Coronary artery disease (%)	7 (13.7)
Renal insufficiency (%)	7 (13.7)
Smoking (%)	30 (58.8)
Gout (%)	5 (9.8)
History of cardiovascular surgery (%)	3 (5.9)
Family history of aortic diseases (%)	1 (2.0)
Accidental identification (%)	22 (32.8)
Red blood cell (10^12/L, mean ± SD)	4.5 ± 0.9 (Range: 2.8, 8.1)
White blood cell (10^9/L, mean ± SD)	9.0 ± 4.4 (Range: 3.7, 28.5)
Platelet (10^9/L, mean ± SD)	243.5 ± 79.2 (Range: 120.0, 599.0)
D-dimer (ng/mL, mean ± SD)	1876.8 ± 2121.3 (Range: 220.0, 8790.0)
Below renal artery (%)	46 (67.6)
Diameter (mm, mean ± SD)	27.6 ± 9.9 (Range: 13.0, 57.0)
False lumen thrombosis (%)	
CT	19 (27.9)
P	25 (36.8)
PT	24 (35.3)
Treatment (%)	
BMT	38 (55.9)
EVAR	27 (39.7)
Open Surgery	3 (4.4)

SD, standard deviation; CT, complete thrombosis; P, patency, PT, partial thrombosis; BMT, best medical treatment; EVAR, endovascular aneurysm repair.

**Table 2:** Baseline characteristics of the conservatively treated cohort.

Variables	Mean ± SD (Range)/Count (Percentage)
*n*	38
Age (year, mean ± SD)	63.4 ± 16.0 (Range: 26.0–93.0)
Male (%)	29 (76.3)
Hypertension (%)	20 (87.0)
Diabetes (%)	2 (8.7)
Hyperlipidimia (%)	4 (17.4)
Chronic obstructive pulmonary disease (%)	5 (21.7)
Coronary artery disease (%)	2 (8.7)
Renal insufficiency (%)	2 (8.7)
Smoking (%)	14 (60.9)
Gout (%)	1 (4.3)
History of cardiovascular surgery (%)	3 (13.0)
Family history of aortic diseases (%)	0 (0)
Accidental identification (%)	12 (32.4)
Red blood cell (10^12/L, mean ± SD)	4.7 ± 1.1 (Range: 2.8, 8.1)
White blood cell (10^9/L, mean ± SD)	9.2 ± 5.1 (Range: 3.7, 28.5)
Platelet (10^9/L, mean ± SD)	260.5 ± 103.7 (Range: 120.0, 599.0)
D-dimer (ng/mL, mean ± SD)	1592.1 ± 2005.9 (Range: 220.0, 8790.0)
Below renal artery (%)	24 (63.2)
Diameter (mm, mean ± SD)	28.5 ± 9.9 (Range: 13.0, 57.0)
False lumen thrombosis (%)	
CT	9 (23.7)
P	15 (39.5)
PT	14 (36.8)

SD, standard deviation; CT, complete thrombosis; P, patency, PT, partial thrombosis; BMT, best medical treatment.

**Figure 2:**
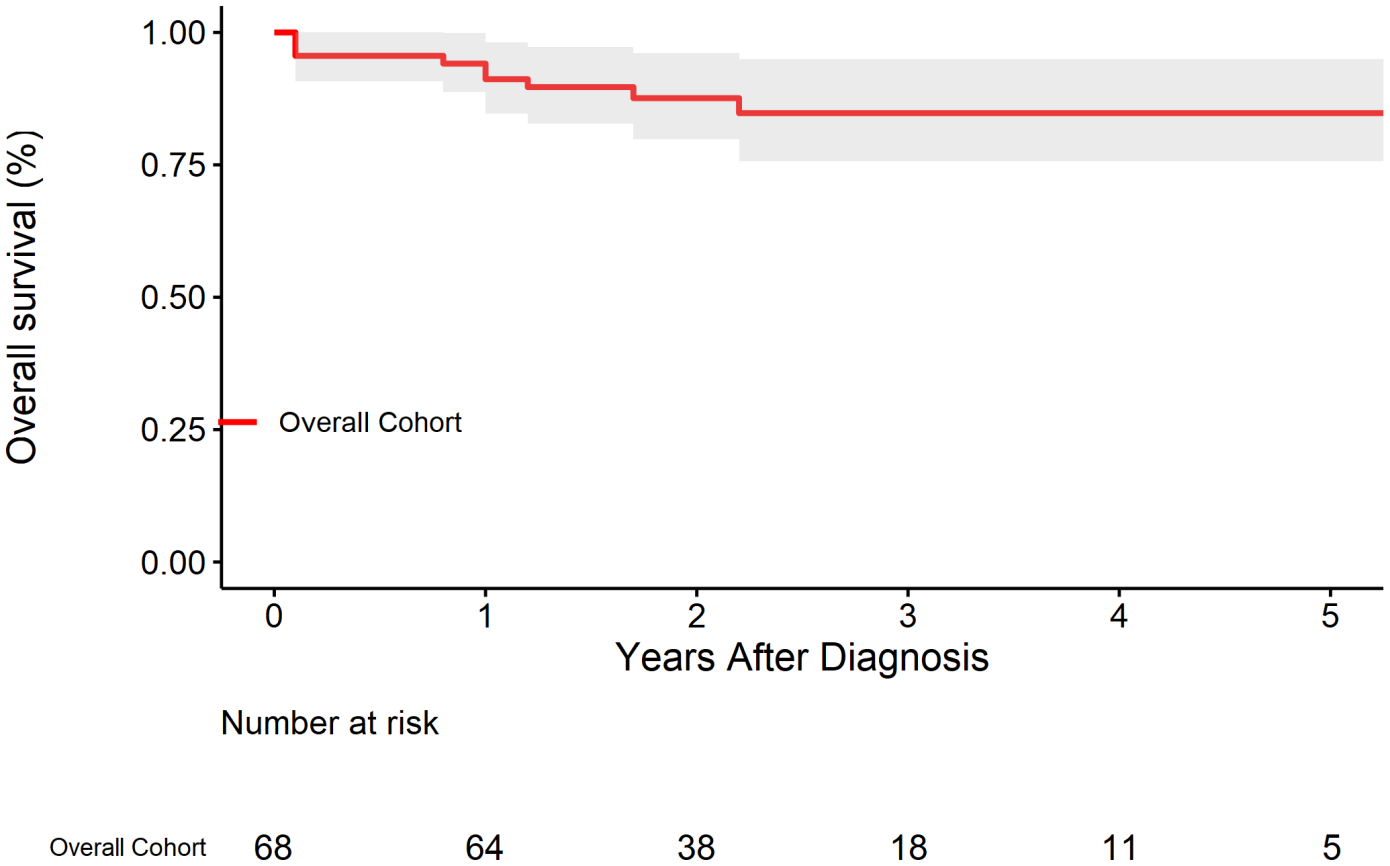
Kaplan–Meier survival curve for the overall cohort.

**Figure 3:**
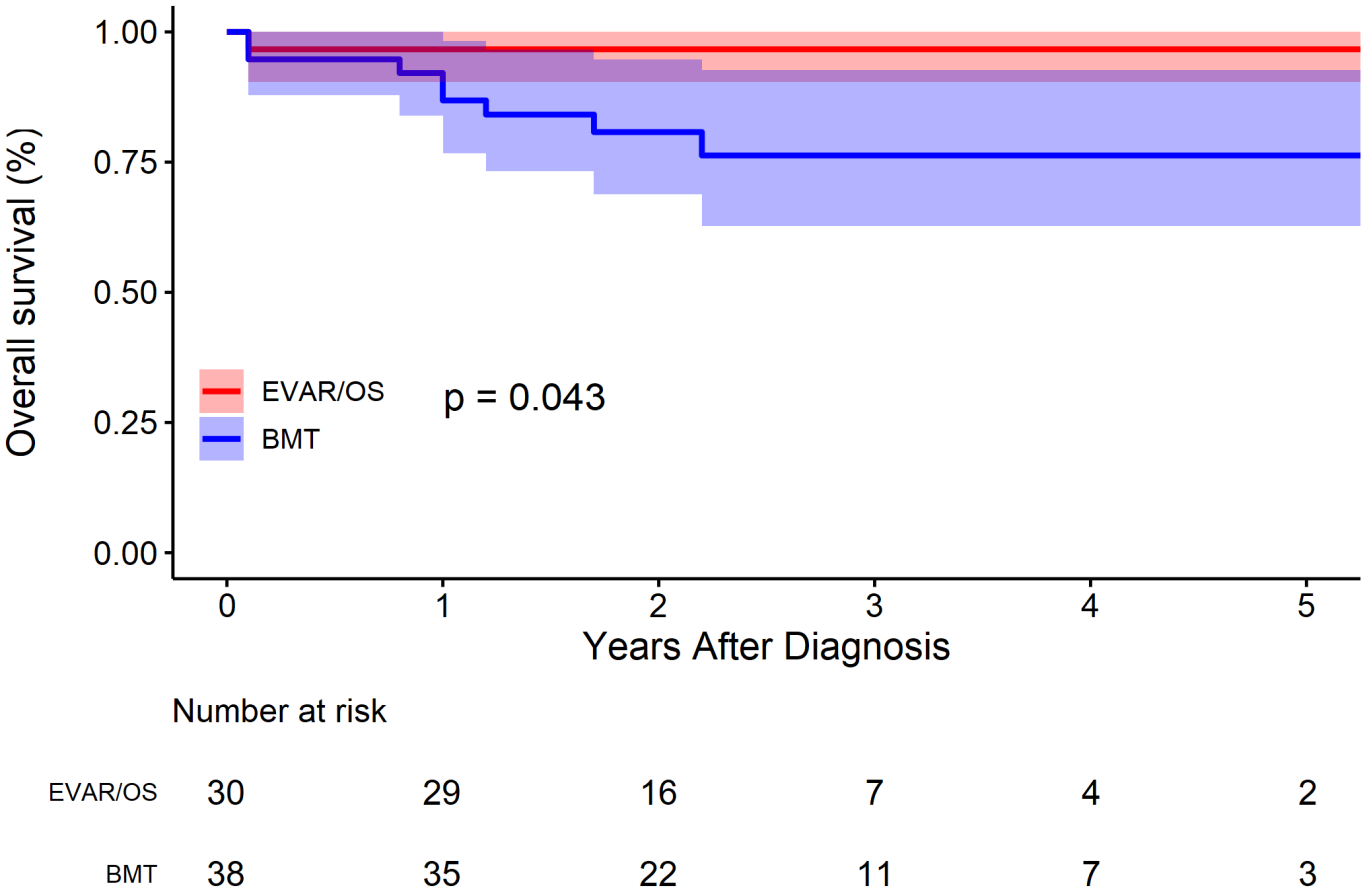
Kaplan–Meier survival curve by treatment for the overall cohort.

The survival rate of the overall cohort was shown in Figure 2. The survival rates at 1, 3, and 5 years were 91.2% (95% CI: 84.7–98.2%), 84.8% (95% CI: 75.7–94.9%), and 84.8% (95% CI: 75.7–94.9%), respectively. As was shown in Figure 3, Compared with EVAR/OS, patient treated conservatively had a worse survival (*p* = 0.043). The survival rates at 1, 3, and 5 years were 96.6% (95% CI: 90.4–100.0%), 96.6% (95% CI: 90.4–100.0%), and 96.6% (95% CI: 90.4–100.0%), respectively for EVAR/OS group, and 86.8% (76.7–98.2%), 76.3 (95% CI: 62.7–92.7%), and 76.3% (95% CI: 62.7–92.7%), respectively for BMT group. The HR of BMT versus EVAR/OS was 6.4 (95% CI: 0.8–51.3, *p* = 0.079). The natural history of the IAAD was then assessed based on the BMT cohort by aortic size, false lumen status, sex, and symptoms, respectively, as follows.

**Figure 4:**
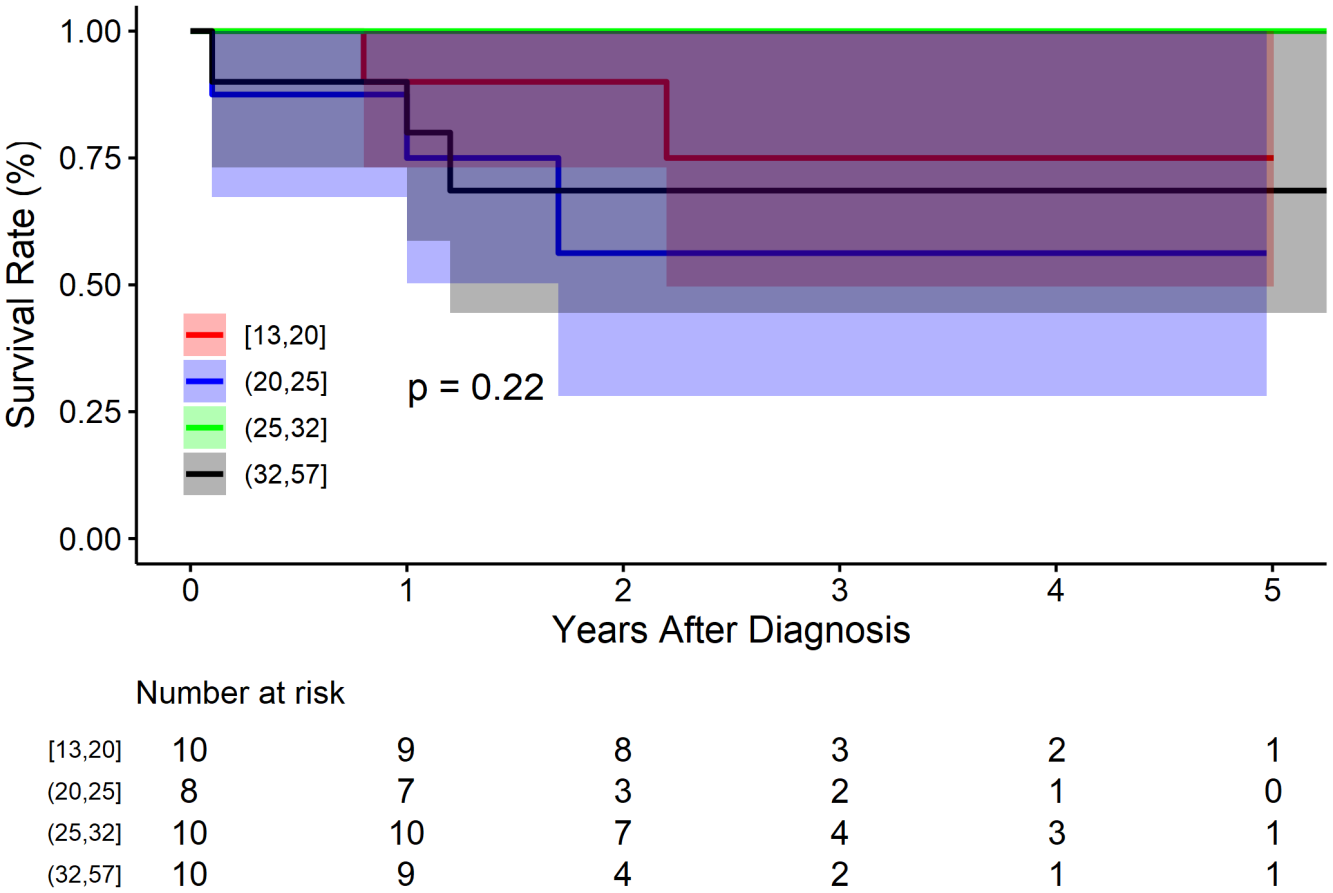
Kaplan–Meier survival curve by aortic size for the conservatively treated cohort.

The BMT cohort was divided into four groups according to the quartiles of diameter: Q1: 13–20 mm (*n* = 10), Q2: 21–25 mm (*n* = 8), 26–32 mm (*n* = 10), 33–57 mm (*n* = 10). Figure 4 shows that there was no significant difference between IAAD of different sizes regarding mortality (*p* = 0.220). The survival rate at 1, 3, and 5 years of follow up were 90.0% (95% CI: 73.2–100.0%), 75.0% (95% CI: 49.6–100.0%), and 75.0% (95% CI: 49.6–100.0%) respectively for Q1 of aortic size, and 75.0% (95% CI: 50.3–100.0%), 56.2% (95% CI: 28.1–100.0%), and 56.2% (95% CI: 28.1–100.0%) respectively for Q2 of aortic size, and 100.0% (95% CI: 100.0–100.0%), 100.0% (95% CI: 100.0–100.0%), and 100.0% (95% CI: 100.0–100.0%) respectively for Q3 of aortic size, and 80.0% (95% CI: 58.7–100.0%), 68.6% (95% CI: 44.5–100.0%), and 68.6% (95% CI: 44.5–100.0%) respectively for Q4 of aortic size. Compared to Q1 of aortic size, the HR of mortality for Q2 and Q4 were 2.2 (95% CI: 0.3–13.6, *p* = 0.372), and 1.7 (95% CI: 0.2–10.7, *p* = 0.526), respectively. The HR for Q3 was not applicable because no events were observed in this sub-cohort.

**Figure 5:**
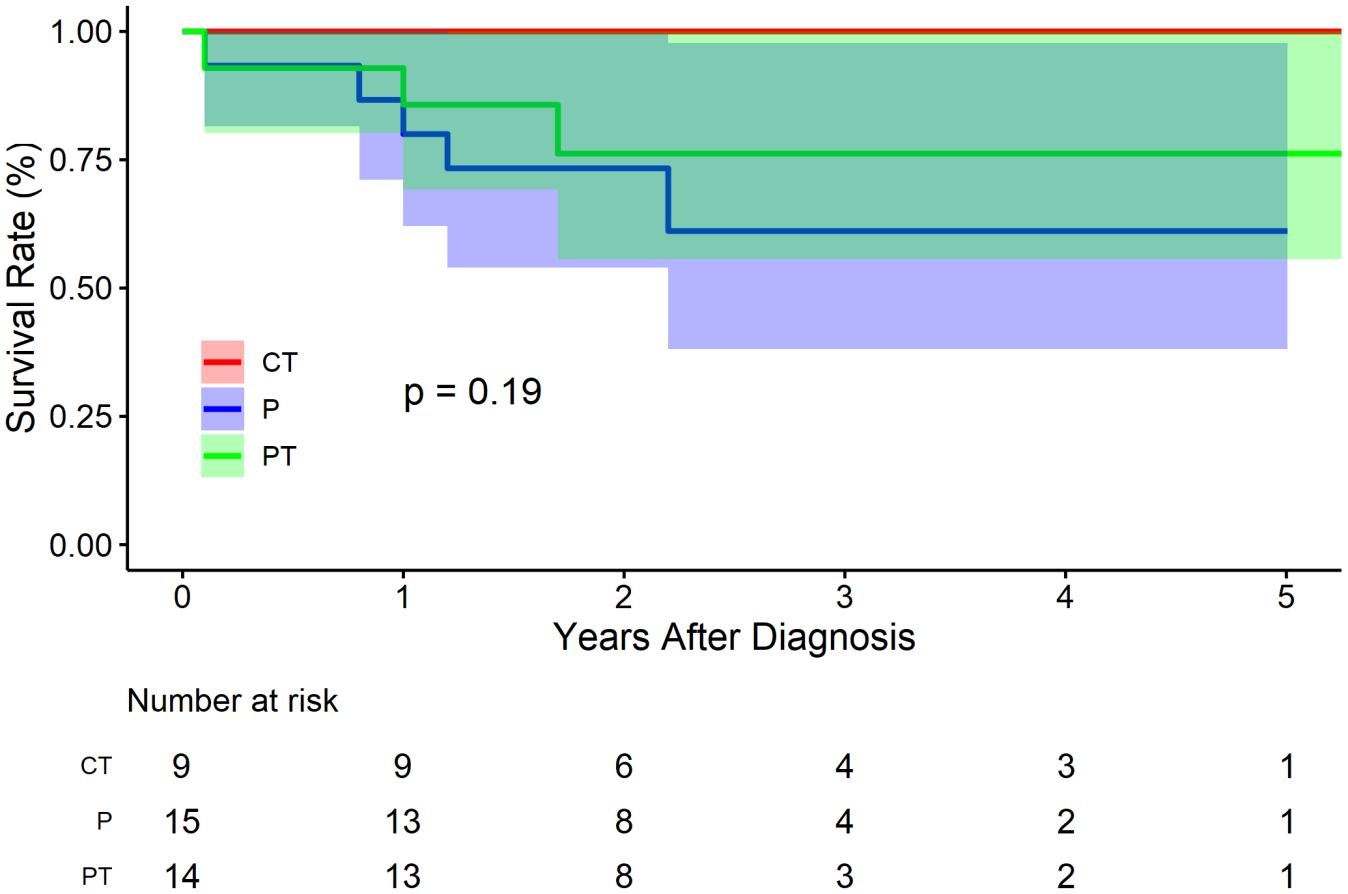
Kaplan–Meier survival curve by false lumen thrombosis status for the conservatively treated cohort. CT, complete thrombosis; PT, partial thrombosis; P, patency.

As regards false lumen status, there were 9 cases of CT, 15 cases of P, and 14 cases of PT in the BMT cohort. Figure 5 shows that CT group fared improved survival rate, followed by group PT and P, respectively, although not significantly (*p* = 0.190). The survival rate at 1, 3, and 5 years of follow up were 100.0% (95% CI: 100.0–100.0%), 100.0% (95% CI: 100.0–100.0%), and 100.0% (95% CI: 100.0–100.0%) respectively for CT group, and 80.0% (95% CI: 62.1–100.0%), 61.1% (95% CI: 38.2–97.8%), and 61.1% (95% CI: 38.2–97.8%) respectively for P group, and 85.7% (95% CI: 69.2–100.0%), 76.2% (95% CI: 55.6–100.0%), and 76.2% (95% CI: 55.6–100.0%) respectively for PT group. Compared to group P, the HR of mortality for group PT was 0.6 (95% CI: 0.1–2.5, *p* = 0.487). The HR for group CT was not applicable because no events were observed in this sub-cohort.

**Figure 6:**
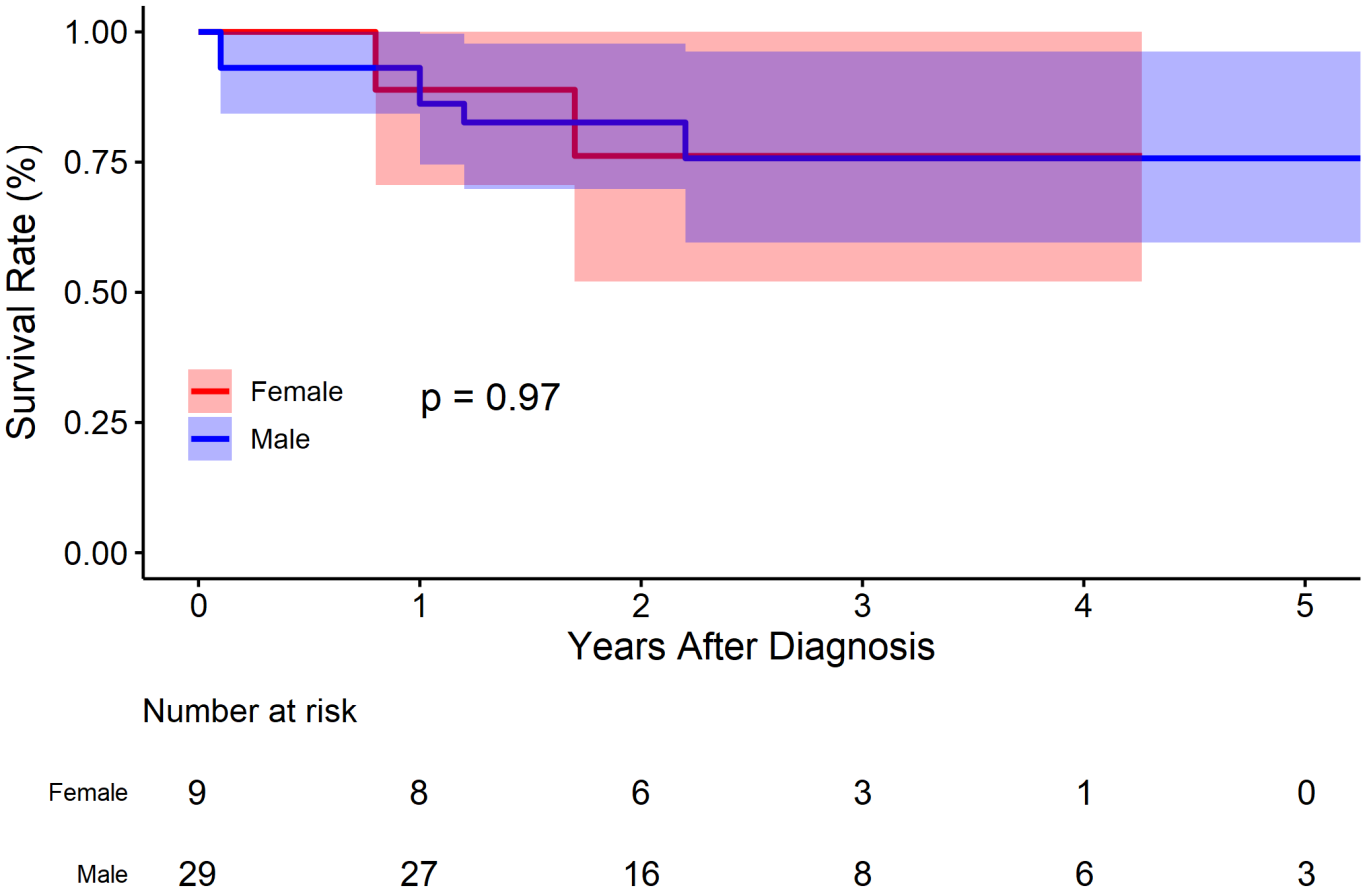
Kaplan–Meier survival curve by sex for the conservatively treated cohort.

There were 29 males and 9 females in the BMT cohort. As was demonstrated in Figure 6, no significant deference was observed between male and female concerning survival rate (*p* = 0.970). The survival rate at 1, 3, and 5 years of follow up were 86.2% (95% CI: 74.5–99.7%), 75.7% (95% CI: 59.6–96.2%), and 75.7% (95% CI: 59.6–96.2%), respectively for male, and 88.9% (95% CI: 70.6–100.0%), 76.2% (95% CI: 52.1–100.0%), and 76.2% (95% CI: 52.1–100.0%) respectively for female. Compared to female, the HR of mortality for male was 1.0 (95% CI: 0.2–5.1, *p* = 0.963).

**Figure 7:**
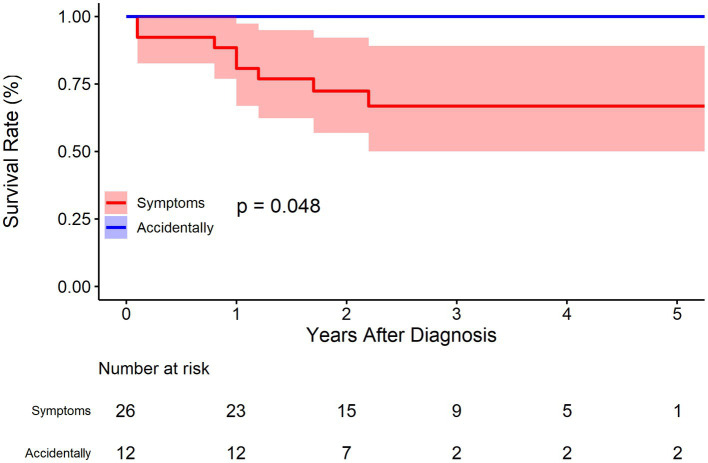
Kaplan–Meier survival curve by symptoms for the conservatively treated cohort.

For symptoms (abdominal pain or others), 12 cases were identified without any symptoms in the BMT cohort. As was demonstrated in Figure 7, patients without symptoms had better outcomes in terms of survival rate (*p* = 0.048). The survival rate at 1, 3, and 5 years of follow up were 80.7% (95% CI: 66.9–97.4%), 66.8% (95% CI: 50.0–89.1%), and 66.8% (95% CI: 50.0–89.1%), respectively for patients with symptoms, and 100.0% (95% CI: 100.0–100.0%), 100.0% (95% CI: 100.0–100.0%), and 100.0% (95% CI: 100.0–100.0%) respectively for patients without symptoms. The HR for patients without symptoms was not applicable because no events were observed in this sub-cohort.

Further, K-M curves (Supplementary Figures 1–4) with mortality and intervention as the combined endpoints were performed, demonstrating similar results as above.

## Discussion

IAAD is a unique and extremely rare aortic disorder. It has long been neglected in almost all the guidelines or consensus for aortic diseases (7–11). Little was known for its natural history, rendering the clinical decision rather difficult. In the present study, we investigated the natural history of IAAD with a prospective cohort, revealing several interesting facts.

Reassuringly, compared to a 5-year survival of 65% for Type B aortic dissection (12), the prognosis of IAAD is pretty good, with survival rates of 91.2% (95% CI: 84.7–98.2%), 84.8% (95% CI: 75.7–94.9%), and 84.8% (75.7–94.9%), at 1, 3, and 5 years, respectively. It was found that patient treated conservatively had a worse survival (*p* = 0.043) compared to surgically intervened patients (either EVAR or OS). Strikingly, the HR of mortality for the BMT group is up to 6 times higher than that of the EVAR/OS group. Of note, only 2 patients received OS, demonstrating the increasing popularity and safety of EVAR, which is consistent to our previous finding that from 2002 to 2018, the prevalence of EVAR for the treatment of IAAD has been increasing, whereas that of OS has been declining (2). Our previous meta-analysis showed better outcomes for conservative treatment, which is contrary to the findings of this study. It is important to note that the patients included in this study were enrolled from 2016 to 2021, when EVAR techniques and devices were supposedly more sophisticated. In contrast, the previous meta-analysis spanned a wide range of years from 1990 to 2018, which may have a temporal bias that cannot be ignored. We speculated that because of the high intervention-related mortality in early days, the treatment regimen tended to be more conservative. Those who received OS or EVAR might have been intrinsically “sicker” and more urgent than conservatively managed patients.

So far, the diameter has always been the cornerstone for aortic aneurysm intervention. A diameter of or above 55.0 mm was recognized as the surgical indication for thoracic aortic aneurysm worldwide, owing to the pioneering work by Elefteriades et al. (13–16). According to the Laplace’s law, wall tension is proportional to the vessel radius for a given blood pressure. Presumably, the larger an aortic aneurysm, the greater risk it will take for aortic dissection. Similarly, the larger an aortic dissection is, the greater risk it will take for rupture/death. However, we found that aortic size did not play a significant a role in the IAAD prognosis, suggesting that aortic size might not be taken as a surgical indication for IAAD. Note that we do not consider IAAD with large diameter to be less dangerous, but rather emphasize that IAAD with small diameter is equally dangerous. And we lack sufficient data for large diameter IAAD. Overall, the median diameter of IAAD was only 27.0 mm, far less than the current surgical recommendation of 55.0 mm. The mean aortic diameter for type A aortic dissection is 53.8 mm (17) and the median aortic size at type B aortic dissection is 41.0 mm (18). It’s interesting to note that aorta dissects at smaller sizes progressively as it extends from ascending, descending to abdominal aorta. Even the fatality of aortic dissection decreases in the same way. And ascending aorta is more prone to dissection (hence the scarcity of ascending aortic rupture), the abdominal aorta is more susceptible to rupture (hence the rarity of IAAD), while the descending aorta behaves in the middle. These fascinating phenomena deserve further investigations.

PT of the false lumen was first brought into attention by Tsai et al. (5). They found that PT at discharge was a strong predictor of mortality in patients with type B aortic dissection compared with CT and P. It was reported that the mean 3-year mortality rate for patients with a patent false lumen was 13.7 ± 7.1%, for those with partial thrombosis was 31.6% ± 12.4, and for those with complete thrombosis was 22.6% ± 22.6 (*p* = 0.003). It was speculated that thrombosis at the distal end of the false lumen may block the secondary entry tear to form a “blind sac” structure, increasing the pressure of the false lumen, or PT may increase the risk of rupture as a result of hypoxia of the arterial wall adjacent to the intraluminal thrombus, which leads to increased local inflammation, neovascularization, and localized wall weakening. However, the current study does not support the negative role of PT on the survival of IAAD. Figure 5 shows the trend that CT group fared improved survival rate, followed by group PT and P, respectively (*p* = 0.190), which is consistent with the clinical instinct that PT was merely a transitional phase between CT and Kudo et al. also found no difference in the survival rate among groups PT, P, and CT. The event-free rate was the greatest in group CT, with a 3-and 5-year event-free rate of 100 and 95.7%, respectively (19). Our recent study also demonstrated no significant differences in aortic growth between the three groups (20). Although no statistical difference was reached, the outcome was much worse in the P group in terms of trend, and an obvious gradient effect on the outcomes between P, PT, and CT sequentially was observed (Figure 5). Thus, the current evidence does not favor PT as a surgical indication for IAAD.

Sex differences in aortic disease is gaining increasing attention. The prevalence of thoracic aortic aneurysm/dissection in men and women is approximately 70%: 30% (21). This study also shows that men are more susceptible to IAAD. Despite the low prevalence of aortic aneurysm, women may endure a higher risk of dissection or rupture (22). Although women seem to have a natural immunity to aortic disease, those who already develop this disease may bear a more severe burden of aortic wall lesions or be exposed to greater hemodynamic stress. Sokolis and colleagues reported higher levels of metaloproteinase-2 and-9 and reduced expression of tissue inhibitor of matrix metaloproteinase-1 and-2 in women than in men. This impairment for aortic wall homeostasis leads to enhanced degradation of the extracellular matrix, increased stiffness and reduced strength (23). Chunget al. found that despite the relatively uncomplicated procedure and shorter cardiopulmonary bypass time compared to men, women had a higher mortality rate (11% versus 7.4%; *p* = 0.02) (24). International Registry of Acute Aortic Dissection (IRAD) also reported that women had an older age of onset, a later diagnosis, a higher incidence of coma, hypotension, and tamponade, and a worse surgical repair (21). Several epidemiological studies highlighting sexual dimorphism in the development and progression of abdominal aortic aneurysm have shown that women are at greater risk for aneurysm rupture and morbidity after surgical repair (25, 26). Data even suggest women are no longer protected from developing abdominal aortic aneurysm after menopause (27). However, current evidence on the effect of gender remain inconsistent. Some other studies have concluded that there is no outcomes difference in thoracic aortic disease between men and women (28, 29). Friedrich et al. argued that gender itself was no risk factor for mortality and the decision-making for surgical treatment should not depend on gender (28). Our data also demonstrated that no significant difference was observed between male and female concerning survival rate (*p* = 0.970) in IAAD. The survival rate at 1, 3, and 5 years of follow up were 86.2% (95% CI: 74.5–99.7%), 75.7% (95% CI: 59.6–96.2%), and 75.7% (95% CI: 59.6–96.2%), respectively for male, and 88.9% (95% CI: 70.6–100.0%), 76.2% (95% CI: 52.1–100.0%), and 76.2% (95% CI: 52.1–100.0%) respectively for female. Compared to female, the HR of mortality for male was 1.0 (95% CI: 0.2–5.1, *p* = 0.963). More in-depth research is needed on this conflicting issue.

In our previous study, symptoms were placed at the core of IAAD management based on literature reports (2). It was recommended that for cases of asymptomatic uncomplicated IAAD, conservative treatment with continuous monitoring and evaluation is preferred. For cases of asymptomatic complicated IAAD, invasive treatment should be considered, with EVAR as first-line therapy, followed by OS. In contrast, those with symptomatic IAAD should first receive conservative treatment and then be assessed if the symptoms persist. If the symptoms subside, the patients should be treated in accordance with the protocol for asymptomatic IAAD. If the symptoms persist, invasive treatment should be undertaken. Of note, the previous recommendations based on literature meta-analysis favored conservative treatment, which is challenged by the updated findings. In consistency to the aforementioned meta-analysis, the present study confirms the important role of symptoms in clinical decision for IAAD. As was demonstrated in Figure 7, patients without symptoms had better outcomes as regards survival rate (*p* = 0.048). The survival rate at 1, 3, and 5 years of follow up were 80.7% (95% CI: 66.9–97.4%), 66.8% (95% CI: 50.0–89.1%), and 66.8% (95% CI: 50.0–89.1%), respectively for patients with symptoms, and 100.0% (95% CI: 100.0–100.0%), 100.0% (95% CI: 100.0–100.0%), and 100.0% (95% CI: 100.0–100.0%) respectively for patients without symptoms.

## Limitations

Our analysis should be interpreted in the context of several limitations. First, this study was based on single-center experience with a high volume of cardiovascular operations. The external validity of our results needs further investigation. Second, we reported a relatively short duration of follow-up. An ongoing follow-up and report are expected. Third, unfortunately, although the sample size of this study was acceptable considering the extreme rarity of IAAD, the statistical power was insufficient for us to perform a multi-variable analysis. Fourth, the exact cause of death cannot be confirmed, despite our best efforts. It may result in some bias.

## Conclusion

On the basis of patients’ preference and surgeons’ experience, a more aggressive treatment regimen for IAAD should be considered, with EVAR being the most likely preferred choice, especially for those with persistent symptoms and patent false lumen, regardless of sex, age, or aortic size.

## Data availability statement

The raw data supporting the conclusions of this article will be made available by the authors, without undue reservation.

## Ethics statement

The studies involving human participants were reviewed and approved by the Institutional Review Board of GDPH. Written informed consent for participation was not required for this study in accordance with the national legislation and the institutional requirements.

## Author contributions

JW had full access to all of the data in the study and takes responsibility for the integrity of the data and the accuracy of the data analysis. JW and YW: concept and design. JW and DZ: drafting of the manuscript. YC and ZC: critical revision of the manuscript for important intellectual content. JY and JL: statistical analysis. XL and TS: obtained funding. RF: administrative, technical, and material support. JW: supervision. JW, YW, FL, DZ, YC, ZC, JY, JL, XL, RF, and TS: acquisition, analysis, or interpretation of data. All authors contributed to the article and approved the submitted version.

## Funding

This work was supported by the National Natural Science Foundation of China (82200518).

## Conflict of interest

The authors declare that the research was conducted in the absence of any commercial or financial relationships that could be construed as a potential conflict of interest.

## Publisher’s note

All claims expressed in this article are solely those of the authors and do not necessarily represent those of their affiliated organizations, or those of the publisher, the editors and the reviewers. Any product that may be evaluated in this article, or claim that may be made by its manufacturer, is not guaranteed or endorsed by the publisher.

## Supplementary material

SUPPLEMENTARY FIGURE S1 Freedom from death and intervention by aortic size for the conservatively treated cohort.

SUPPLEMENTARY FIGURE S2 Freedom from death and intervention by aortic size for the conservatively treated cohort. CT, complete thrombosis; PT, partial thrombosis; P, patency.

SUPPLEMENTARY FIGURE S3 Freedom from death and intervention by sex for the conservatively treated cohort.

SUPPLEMENTARY FIGURE S4 Freedom from death and intervention by symptoms for the conservatively treated cohort.
