# Supplementary figures and images for: Natural history of isolated abdominal aortic dissection: A prospective cohort study
Source: Front Cardiovasc Med. 2023 Feb 23;10:1002832. doi: 10.3389/fcvm.2023.1002832 (PMC9996307; doi:10.3389/fcvm.2023.1002832)

Supplemental Figure 1


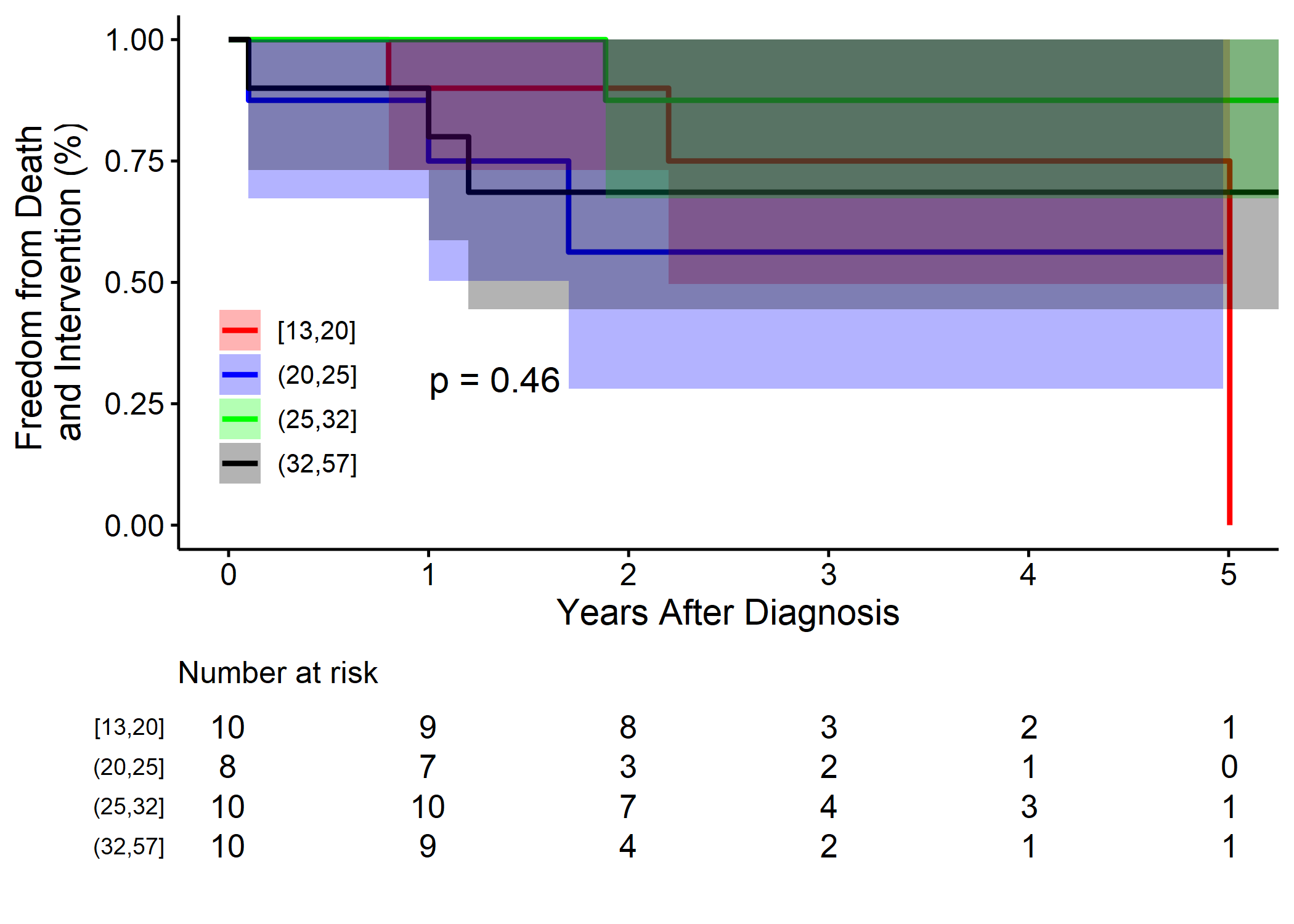


Supplemental Figure 2


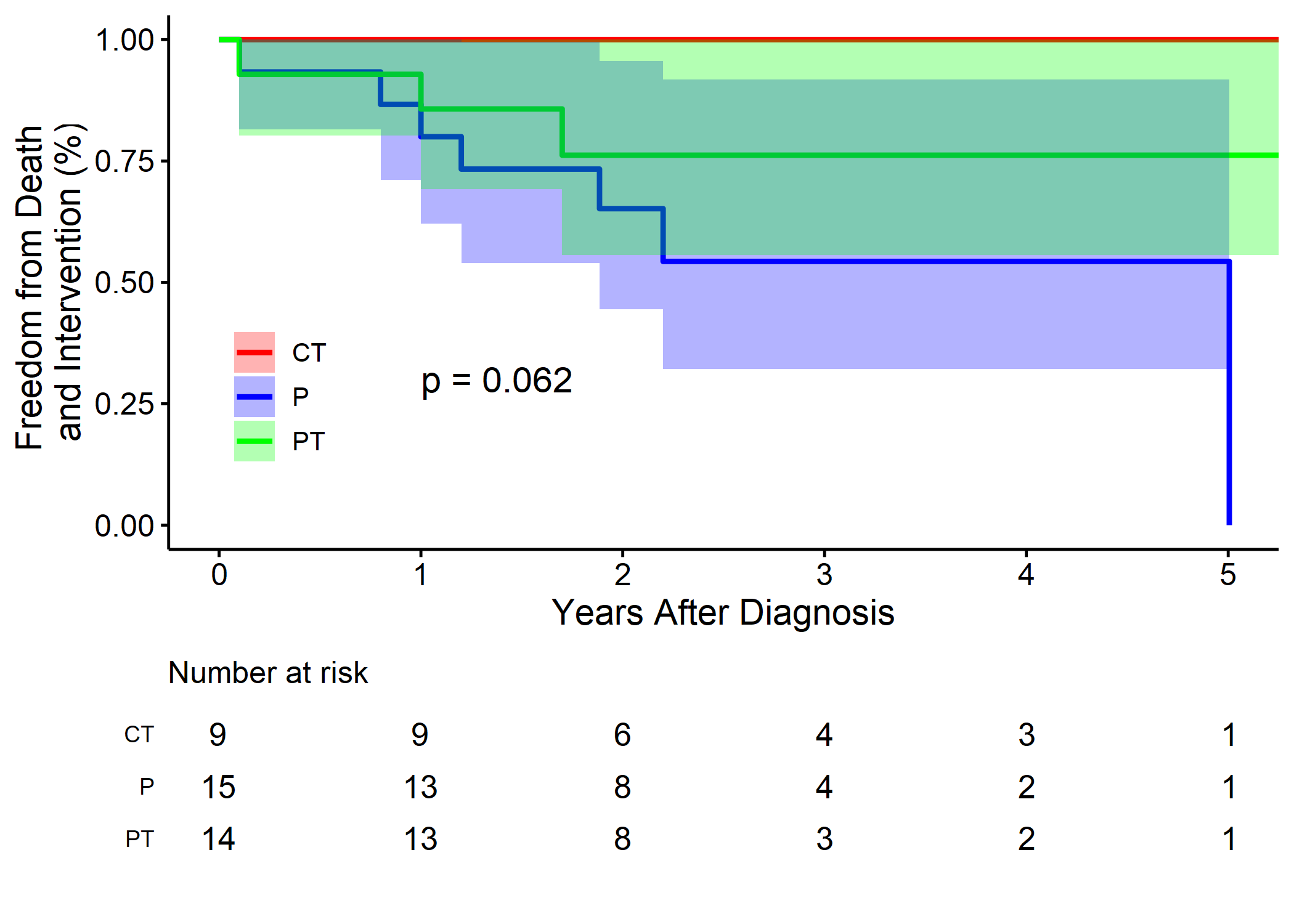


Supplemental Figure 3


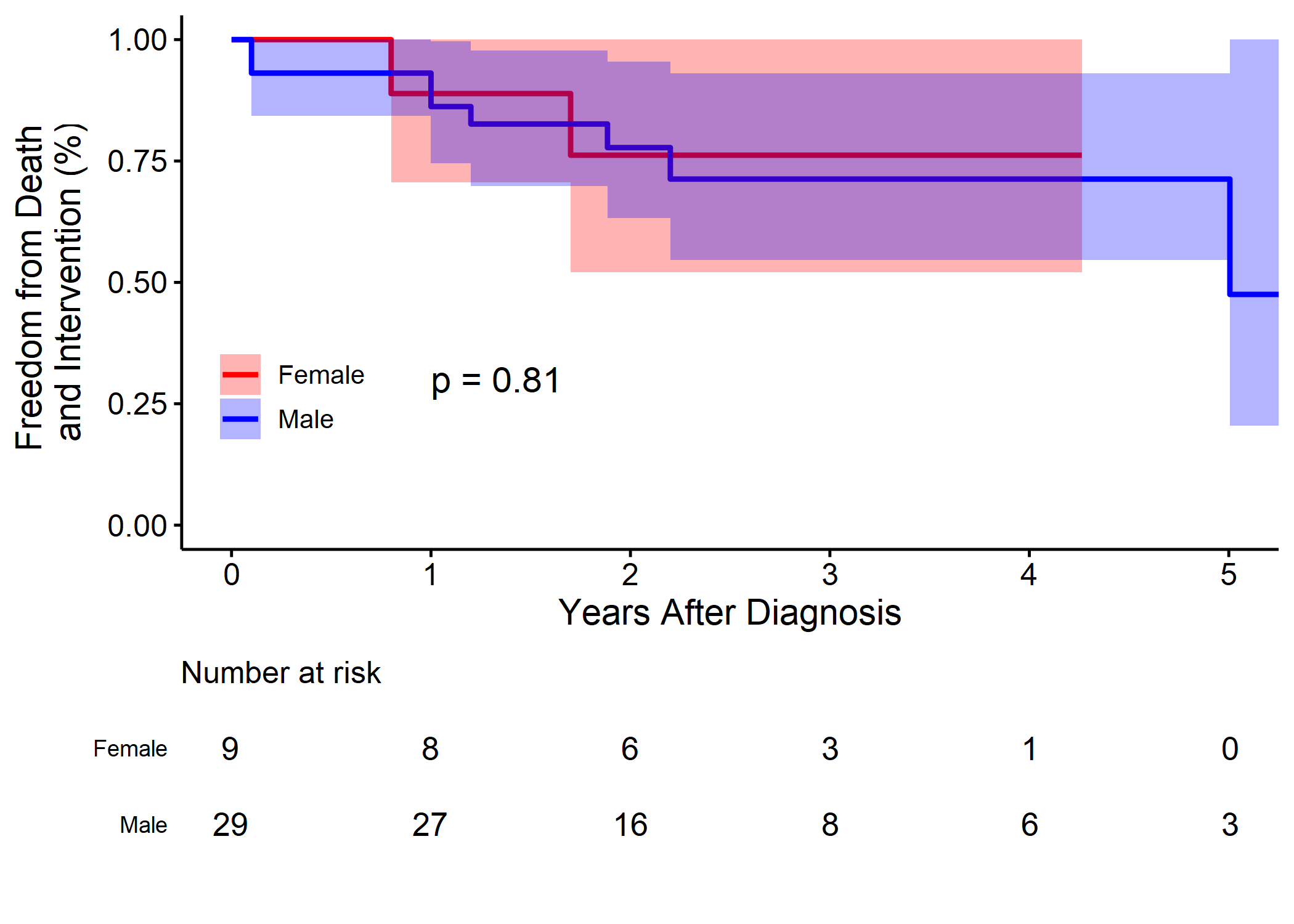


Supplemental Figure 4


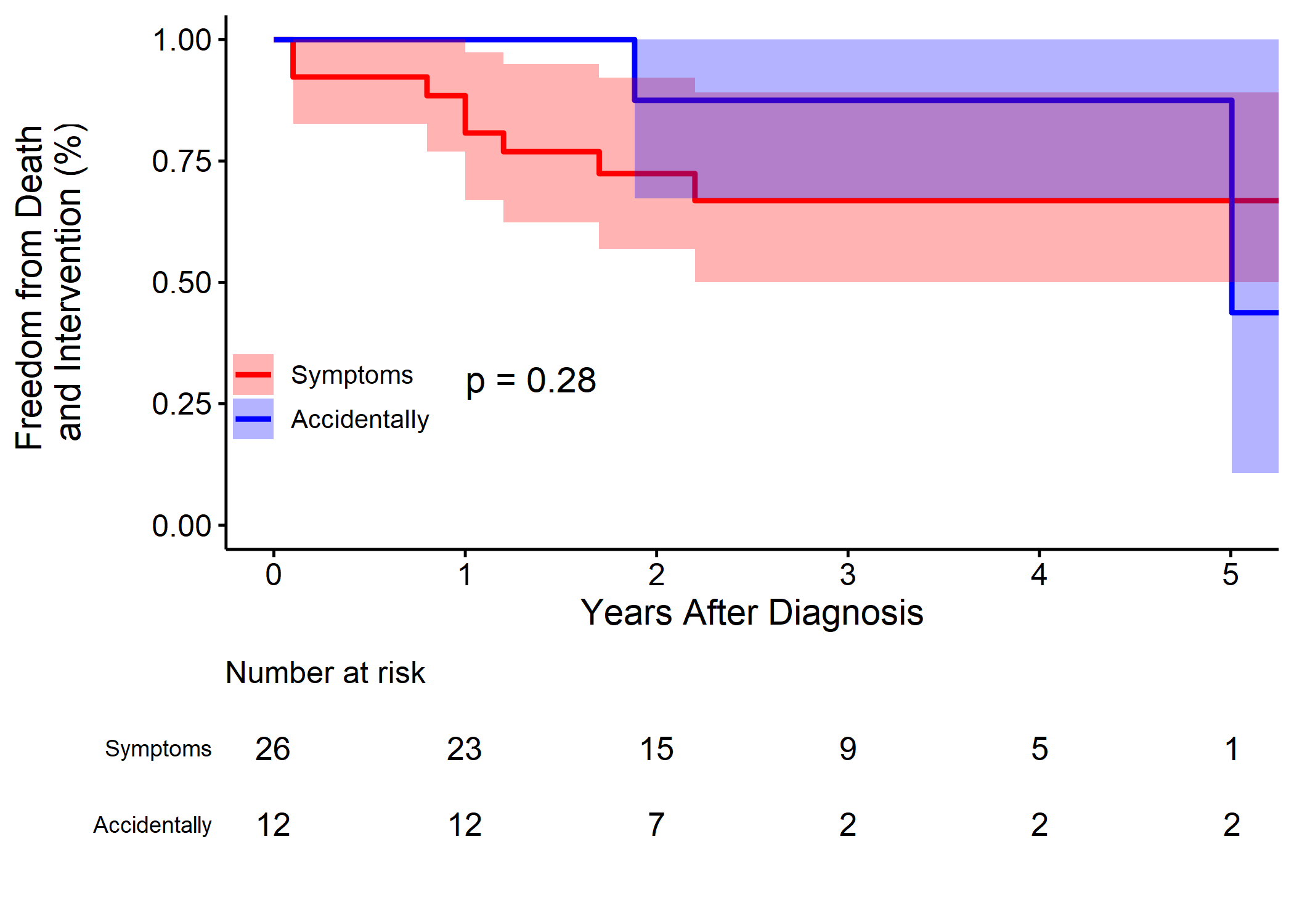

Supplement: SUPPLEMENTARY FIGURE S1 — Freedom from death and intervention by aortic size for the conservatively treated cohort. [file Table_1.DOCX]
